# The Patient's Point of View

**Published:** 1910-02-12

**Authors:** Eva Richmond

**Affiliations:** Rockhampton, Falfield, Glos


### Editor's Letter-Box.

### THE PATIENT'S POINT OF VIEW.

*To the Editor of* The Hospital.

Sir,—I have read your note on the patients' opinion of students' demonstrations in hospitals. A man from our country parish was sent into a hospital, suffering from an obscure complaint in his foot. He was used as an "object lesson" to the students, and he proudly told his wife that he had had "the doctors" round his bed, all trying to find out what was the matter with him. He was quite elated at the attention shown him, felt that he was an object of interest, and that the doctors were doing all they could for him.

Another parishioner—a woman—went into the same hospital to see if anything could be done for a Pott's fracture which had set badly. When she returned, she told me her experiences, and described how, when her leg was *x*-rayed, the doctor had explained to the students, by what they saw there, how the fracture should have been set. "Did you mind their being shown your leg?" I asked, knowing the nonsense some people talk about these necessary demonstrations. "No," she replied, "I didn't mind what they did as long as they did me any good." Unfortunately she was too old for the breaking and resetting which would have improved matters; the most that could be done was massage of the limb and the supplying of an iron for wear in the boot.—Yours faithfully,

Eva Richmond.

Rockhampton, Falfield, Glos.

